# Distinct response patterns of endothelial markers to the BNT162b2 mRNA COVID-19 booster vaccine are associated with the spike-specific IgG antibody production

**DOI:** 10.3389/fimmu.2024.1471401

**Published:** 2025-01-06

**Authors:** Beatriz Castro-Robles, Francisco J. Cimas, Lourdes Arias-Salazar, Jesús Ontañón, Julia Lozano, Susana López-López, Fernando Andrés-Pretel, María Ángeles Requena-Calleja, Antonio Mas, Gemma Serrano-Heras, Tomás Segura, Javier Solera

**Affiliations:** ^1^ Research Unit, General University Hospital of Albacete, Health Service of Castilla-La Mancha (SESCAM), Albacete, Spain; ^2^ Molecular Oncology Laboratory, Molecular Medicine Unit, Associated Unit of Biomedicine, University of Castilla-La Mancha-Spanish National Research Council (UCLM-CSIC), Albacete, Spain; ^3^ Mecenazgo COVID-19, Faculty of Medicine, University of Castilla-La Mancha, Albacete, Spain; ^4^ Immunology Unit, Clinical Analysis Department, General University Hospital of Albacete, Albacete, Spain; ^5^ Microbiology Department, General University Hospital of Albacete, Albacete, Spain; ^6^ Research Unit, General University Hospital of Albacete, Department of Statistics, Foundation of the National Paraplegics Hospital of Toledo, Albacete, Spain; ^7^ Internal Medicine Department, General University Hospital of Albacete, Albacete, Spain; ^8^ Biomedicine Institute of UCLM (IB-UCLM), Faculty of Medicine, University of Castilla-La Mancha, Albacete, Spain; ^9^ Faculty of Pharmacy, University of Castilla-La Mancha, Albacete, Spain; ^10^ Associated Unit of Biomedicine UCLM-CSIC, University of Castilla-La Mancha, Ciudad Real, Spain; ^11^ Neurology Department, General University Hospital of Albacete, SESCAM, Albacete, Spain; ^12^ Faculty of Medicine, University of Castilla-La Mancha, Albacete, Spain

**Keywords:** mRNA COVID-19 vaccines, booster dose, SARS-CoV-2, anti-spike antibodies, IgG, endothelial dysfunction, endocan, VCAM-1

## Abstract

**Introduction:**

Despite the efficacy and safety of SARS-CoV-2 vaccines, inflammatory and/or thrombotic episodes have been reported. Since the impact of COVID-19 vaccines on the endothelium remains uncertain, our objective was to assess endothelial activation status before and 90 days after the third dose of the BNT162b2 mRNA COVID-19 vaccine.

**Methods:**

A prospective longitudinal study was conducted at University General Hospital of Albacete, involving 38 healthy health-care workers. Serum levels of endothelial markers (endocan and sVCAM-1) and spike S1-specific IgG antibodies were determined before and at 7, 15, 24 and 90days following vaccination. To analyze each participant´s individual response, we calculated relative increases/decreases (delta values) in endothelial markers and antibodies concentrations compared to their pre-vaccination levels.

**Results:**

We identified two significantly distinct profiles of endothelial markers response, characterized by either increased or decreased serum levels of endocan and sVCAM. Incremental and decremental response groups did not differ in terms of age, sex, cardiovascular risk factors, previous SARS-CoV-2 infection and influenza vaccine co-administration. However, these responses were significantly associated with the relative spike-specific antibody production. Specifically, the greatest relative increase in antibodies was found in the decremental responders. Additionally, the higher delta antibody production was observed in non-previously infected individuals

**Conclusion:**

Administration of the BNT162b2 booster vaccine triggered a non-homogenous response of endothelial function markers among the study participants. Our findings improve the understanding of individual responses to the mRNA COVID-19 booster vaccine, which could be useful in assessing the need for booster doses, particularly in population at risk of vascular complications.

## 1 Introduction

The development of COVID-19 vaccines has been a great tool in the fight against SARS-CoV-2 infection (1), although some side effects, such as severe inflammatory reactions and thrombotic episodes, have been occasionally documented (2–6). These complications have also been observed during SARS-CoV-2 infection, mainly due to the characteristic hyperinflammatory and procoagulant state of COVID-19, which involves the endothelium both as an effector contributing to inflammation and thrombosis and as target organ (7). Regarding adverse effects of COVID-19 vaccines, a transient deterioration of endothelial function and a temporal deregulation of the coagulation have been proposed as main triggers of vaccination-mediated complications (8, 9).

While COVID-19 vaccines have been widely administrated in recent years, their impact on endothelial function is not well defined. Several studies performed on individuals who received first/second doses of mRNA or adenoviral-vector based COVID-19 vaccines revealed a prominent increase in endothelial inflammatory markers (8, 10) and a temporary decline in endothelial function (9) after vaccination. As far as we know, the potential impact on the endothelium of administering a booster dose after having previously received two doses of the SARS-CoV-2 vaccine has not yet been addressed. In this context, the present study aimed to assess the effect of the third dose of the Pfizer—BioNTech mRNA COVID-19 vaccine (BNT162b2), administered to health-care workers, on the vascular endothelium. For this purpose, we selected endocan (a glycocalyx proteoglycan also known as endothelial cell-specific molecule-1) and sVCAM-1 (soluble form of vascular cell adhesion molecule 1), which are released by endothelial cells during inflammatory responses and/or after injury and have long been considered useful biomarkers in various pathologies (11–17). Serum levels of these endothelial markers were determined, as a measure of endothelial activation status, before and 7, 15, 24 and 90 days after administration of booster vaccine.

## 2 Materials and methods

### 2.1 Study population

We designed a prospective longitudinal study based on the previously reported cohort of healthy healthcare from the University General Hospital of Albacete (18). The study was conducted in accordance with the Declaration of Helsinki and was approved by the local Clinical Ethics Committee (Internal code: 2021-12 EOm). Written informed consent was obtained from all participants.

Main demographic and clinical data, such as age, sex, cardiovascular risk factors, previous vaccination (type of vaccine and time elapsed since the last dose), history of prior COVID-19, and pharmacological treatment before vaccination and during the 90-day follow-up period were registered. Subjects with autoimmune, tumorous, or inflammatory diseases were excluded.

**Figure 1:**
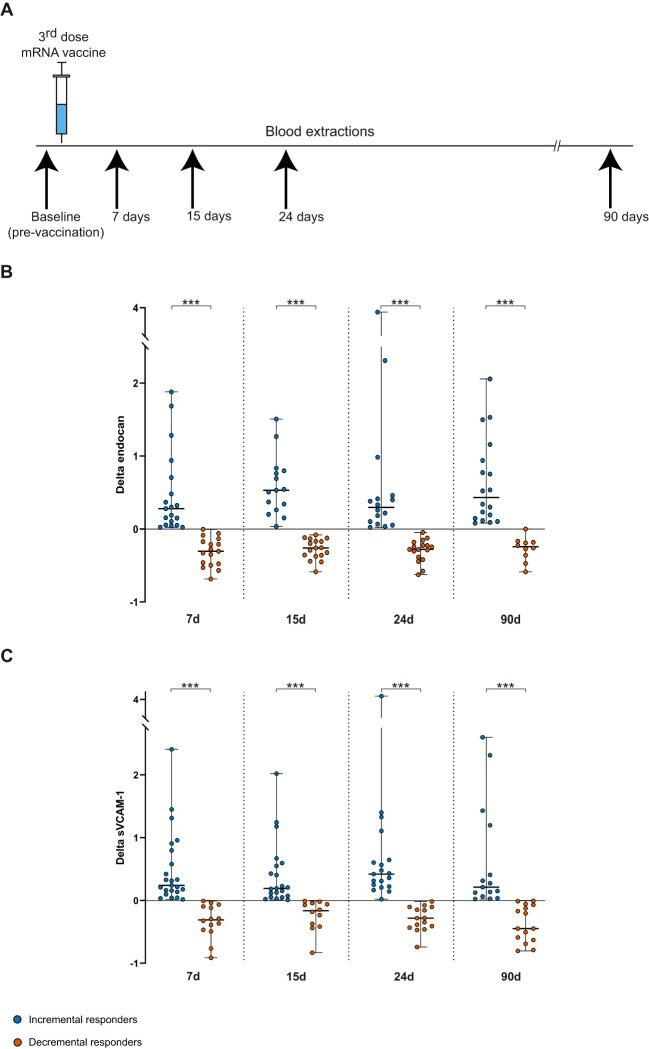
**(A)** An overview of blood sampling time points before and after the booster dose. **(B, C)** Delta [(post-booster value - baseline value/ baseline value)] median (IQR) and individual values of serum endocan and sVCAM-1 levels, respectively, at 7, 15, 24 and 90 days post-vaccination in the incremental (blue dots) and decremental (orange dots) response group. Student's t or Wilcoxon-Mann-Whitney's tests. ***p<0.001.

Participants were invited to provide blood samples before and after vaccination with the booster dose (third dose) of Pfizer—BioNTech COVID-19 vaccine (BNT162b2). It is an mRNA-based vaccine designed for active immunization to prevent COVID-19 caused by SARS-CoV-2 (see **Figure 1A** for blood collection schedule). Of note, all subjects had received the complete vaccination regimen against COVID-19 (first and second doses of BNT162b2 vaccine) 10 months prior to the start of the study.

### 2.2 Determination of serum levels of endothelial markers and anti-spike IgG antibodies

Blood samples were collected at several time-points (pre- and 7, 15, 24 and 90 days post-booster mRNA booster vaccine) from each subject in serum clot activator tube and spun down at 1500x g for 7 minutes at room temperature to separate serum from blood cells. Then, serum was stored at -80°C until analysis of endothelial markers. Serum concentrations of endocan and sVCAM-1 were measured using an enzyme-linked immunosorbent assay kit (ELISA) (Human ESM1 ELISA Kit [Endocan], Abcam, ab213776; Human VCAM-1 ELISA Kit, Sigma-Aldrich, RAB0505), according to the manufacturer’s instructions. Our preliminary ELISA runs indicated that serum samples diluted 1:10 were usually in the range of the standard curve. Standard curve was generated by fitting the data to a 4-parameter logistic (4PL) curve using Arigo Biolaboratories free ELISA calculator at https://www.arigobio.com/elisa-analysis. All samples and standards were assayed in duplicate.

Serum levels of total IgG antibodies against the S1 subunit of the SARS-CoV-2 spike protein that binds to the receptor binding domain (RBD) were measured pre- and post-vaccination using the SARS-CoV-2 IgG II Quantimmunoassay in the Alinity i-System (Abbott, Abbott Park, IL, USA). For the analysis of the data, the antibody units (AU/mL) measured by this immunoassay were converted to standardized WHO binding antibody units (BAU/mL) using the provided conversion factor (AU/mL × 0.142 = BAU/mL). More details are provided in Cimas et al. (19).

### 2.3 Data processing and statistical analysis

In addition to the cohort-wide analysis of serum marker levels (endocan and sVCAM-1), a detailed intra-patient study was performed to investigate each participant’s individual response of endothelial markers and anti-spike IgG antibodies production to the booster dose. To this end, we calculated delta values (relative increases or decreases) to assess variations in the concentration of endothelial function markers and specific IgG antibodies relative to baseline levels. The delta value was calculated as follows:


$$Delta=\;\frac{follow‐up\;value-baseline\;value}{baseline\;values}$$

Baseline values correspond to pre-vaccination values, and follow-up values are described as days 7, 15, 24 and 90 after booster dose. Based on the relative changes (marker delta value) observed, we classified participants into two groups: incremental responders, exhibiting delta values above zero, and decremental responders, showing delta values below zero. We described numeric variables using mean and standard deviation for those that followed normal distributions and median with the inter-quartile range (IQR) for those that did not. Normality was tested using Shapiro-Wilk’s test. The categorical variables were described using frequencies and percentages. Proportion comparisons were conducted using Fisher’s exact test since expected values in the contingency tables were below 5 in all cases.

The differences in means (or medians) were compared using Student’s t test or Wilcoxon-Mann-Whitney test if any of the samples did not follow a normal distribution. To assess association between numeric variables we used Spearman’s correlation. In addition, a multivariate analysis was performed using binary logistic regression to determine whether incremental o decremental responses of endothelial markers could be predicted on the basis of independent variables, including cardiovascular risk factors, previous COVID-19, simultaneous influenza vaccination and delta IgG antibody levels. The regression model was developed using both backward and forward stepwise selection methods, with the likelihood-ratio test employed to identify variables for removal and to refine the model by retaining the most relevant factors. All tests were conducted with an alpha value of 0.05, and 95% confidence interval was calculated where appropriate. Statistical analysis was performed using the R (v4.3.0) software package, and GraphPad Prism (version 8.0.2) was used to create the graphs.

## 3 Results

### 3.1 Endothelial markers and anti-spike IgG antibody responses to mRNA COVID-19 booster dose

The study included a total of 38 individuals (mean age: 48.11 ± 10.77 years; 79% women) who received the third dose of the BNT162b2 mRNA vaccine, administered 10 months after the second dose. The number of subjects with cardiovascular risk factors and those on medication that could affect endothelial marker expression was limited. Approximately half of the participants had a history of SARS-CoV-2 infection at least one year prior to booster vaccine administration. Detailed and additional clinical information is provided in **Supplementary Table S1**. Blood sampling and serum marker concentrations were successfully obtained from 76.3% of all vaccinated participants within 90 days following vaccination (**Table 1**). The median serum values of endocan and sVCAM-1 at baseline were 140.6 pg/ml (IQR: 97.27-189.2) and 99.96 ng/ml (IQR: 69.85-128.7), respectively. Endocan levels showed a mild increase and remained elevated throughout the 90-day follow-up period after the booster vaccine, while sVCAM-1 levels initially rose similarly to endocan levels during the 7-15 days interval but subsequently decreased to near baseline levels (**Table 1**). Analysis of the whole cohort of participants did not reveal statistically significant differences between baseline and follow-up post-vaccination values.

**Table 1:** Serological endothelial markers and total IgG against SARS-CoV-2 spike protein (S1) concentrations.

	Endocan (pg/ml), n	VCAM-1 (ng/ml), n	Total IgG against S1 (BAU/ml), n
**Baseline**	140.6 [97.27-189.2], 37	99.96 [69.8 5-128.7], 38	171.536 [65.4-593.6], 38
**7d**	144.2 [89.31-210.4], 37	113.7 [75.95-137.6], 37	2966.806 [1654.3-5133.9], 35
**15d**	152.5 [120.9-225.5], 33	112.2 [83.46-161], 33	3972.308 [2664.2-5288.9], 38
**24d**	146 [106.6-203.3], 34	104.8 [86.81-140.4], 34	3219.992 [1817.6-3923.7], 27
**90d**	192.6 [126.8-228.3], 29	86.3 [69.46-113.2], 30	2171.89 [869.5-4043.6], 38

Baseline: pre- third dose vaccination; 7d: 7 days post- third dose vaccination; 15d: 15 days post- third dose vaccination; 24d: 24 days post- third dose vaccination; 90d: 90 days post- third dose vaccination; n: analyzed subjects. BAU/ml: Binding Antibody Units per milliliter.

Furthermore, a detailed intra-patient analysis was conducted to assess the response of endothelial markers to the third dose within each individual participant, as outlined in the Materials and Methods section. To describe changes in endocan and sVCAM-1 levels over the 90-day period post-vaccination, we calculated the relative change (delta) in post-vaccine concentrations compared to baseline values. Notably, two significantly distinct patterns of endothelial marker response were observed from 7 to 90 days after mRNA booster vaccine administration. Consequently, vaccinated individuals were categorized into two groups: incremental responders, who showed an increase in marker delta values, and decremental responders, who exhibited a decline in marker delta values. The delta values of serum endocan and sVCAM-1 at days 7, 15, 24 and 90 for each response group are shown in **Figures 1B, C**. Specifically, the serum levels changes in the increased response groups ranged from medians of 0.28 to 0.53 for endocan and from 0.19 to 0.41 for sVCAM-1. The highest increases for each endothelial marker were observed at 15 days and 24 days post-booster vaccine, respectively. In contrast, the group with a reduced endocan response exhibited negative delta values of approximately -0.3 in all measurements after vaccination (**Figure 1B**), whereas participants with a diminished response of sVCAM-1 reached minor peaks of -0.31 and -0.45 at 7d and 90d, respectively, with the smallest decrease detected at 15 days (-0.16) following vaccination (**Figure 1C**). Overall, we observed that more than half of the subjects experienced simultaneous increases (34.29%) or decreases (25.71%) in the post-vaccine levels of both biomarkers. The remaining participants showed a mixed response, with a higher proportion showing reduced endocan levels and increased sVCAM-1 levels (28.57%), compared to those with elevated endocan and decreased sVCAM-1 concentrations (11.43%).

Both incremental and decremental response groups of endocan were homogeneous with respect to age, sex, cardiovascular risk factors, and co-administration of COVID-19 and influenza vaccines within 90 days post-vaccination. Neither group showed statistically significant differences between previously SARS-CoV-2 infected and non-infected subjects (**Supplementary Table S2**). Similarly, sVCAM-1 groups did not show differences in relation to age, sex, cardiovascular risk factors, and co-administration of COVID-19 and influenza vaccines, nor were there statistically significant differences found between previously SARS-CoV-2 infected and non-infected subjects (**Supplementary Table S2**).

**Figure 2:**
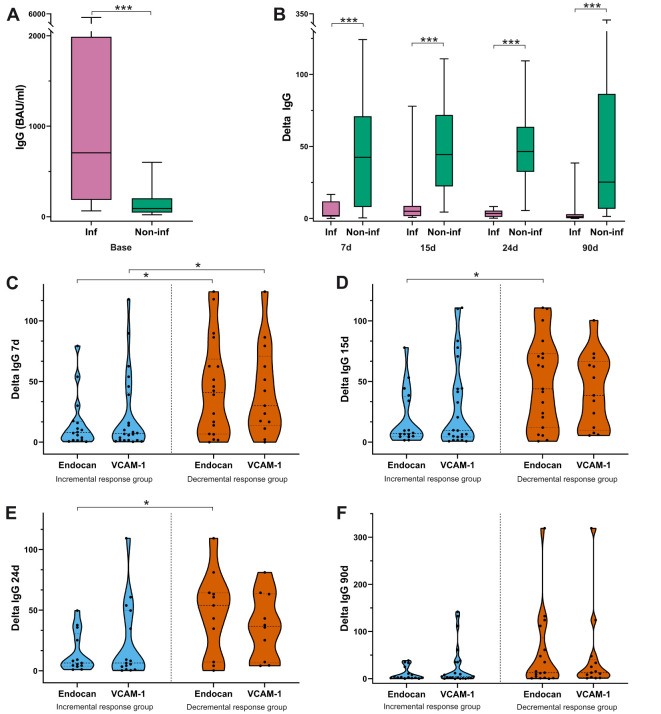
**(A)** Baseline concentration (BAU/ml) of spike-specific IgG antibodies in participants with and without a prior history of COVID-19. **(B)** Delta median (IQR) of anti-spike IgG antibody production at post-vaccination time points for individuals with and without previous SARS-CoV-2 infection. **(C–F)** Relative increase (median-IQR and individual values) in IgG antibody titers in both incremental and decremental responders for endothelial markers from 7 to 90 days after the third dose. Inf: previous SARS-CoV-2 infection; Non-inf: without previous SARS-CoV-2 infection. Student's t or Wilcoxon-Mann-Whitney's tests. *p<0.05; ***p<0.001.

Additionally, our study involved the determination of spike-specific IgG antibody levels before and after the third vaccination dose to measure the extent to which this vaccination, administered 10 months after a second dose, induces antibody responses against SARS-CoV-2-S1 RBD. As shown in **Table 1**, the booster dose produced a remarkable increase in the IgG antibody levels relative to baseline value (171.536 [65.4-593.6] BAU/ml) in all participants. The greatest relative rise in IgG antibody levels was observed within the first 15 days after vaccination. Specifically, the increases (median [IQR]) of circulating antibodies on days 7, 15, 24 and 90 post-mRNA third dose were 13.81 [45.79], 16.25 [55.07], 8.174 [42.7] and 6.407 [33.31], respectively, regarding pre-booster levels. These relative increases in specific-IgG antibodies observed at various intervals post-vaccination (delta at 7, 15, 24, and 90 days) were independent of age, sex, cardiovascular risk factors and concurrent administration of the third anti-SARS-CoV-2 dose and the influenza vaccine. However, these changes were notably influenced by the previous COVID-19 status of participant. Notably, participants with a history of SARS-CoV-2 infection had higher initial concentrations of anti-SARS-CoV-2 IgG antibodies (pre-vaccination; **Figure 2A**, p < 0.001), while those without prior infection exhibited a greater relative increase in IgG antibody production after vaccination (**Figure 2B**, p < 0.001).

### 3.2 Association between serum levels of endocan and sVCAM-1 and specific IgG antibody production after booster vaccination

We next analyzed the potential influence of humoral immunity on the differential response of endothelial markers to the third dose of COVID-19 vaccine. As mentioned above, the administration of the third dose led to significantly increased IgG antibody levels compared to baseline in all participants. However, incremental and decremental response groups for each marker exhibited differences in anti-spike IgG antibody production after the booster compared to pre-vaccination levels. Interestingly, individuals exhibiting reduced levels of endocan and sVCAM-1 were those that showed highest IgG antibody increases during the first month following vaccination. Specifically, we found that administration of the third vaccine dose resulted in a 6.3-7.8 and 6.3-9.5-fold elevation in specific IgG antibodies in the groups showing augmented responses of endocan and sVCAM-1, respectively. The delta values for the decremental response groups for these endothelial markers were remarkable higher, ranging from 40 to 54-fold for endocan, and from 30 to 38.5-fold for sVCAM-1 within the first 24d post-vaccine injection (**Figures 2C–F**, **Supplementary Table S3**).

Differences in the specific IgG antibodies production between the two endocan response groups remained statistically significant at 7, 15 and 24 days post-booster mRNA vaccine. On day 90, such variations in antibody levels between groups were also observed, but they were less notable and did not reach statistical significance (**Figure 2F**, **Supplementary Table S3**). Similar to endocan, the groups with reduced and increased responses of sVCAM-1, showed differences in the relative IgG antibody increase during the first 24 days post-vaccination, but these differences were only statistically significant on day 7 (**Figure 2C**). Furthermore, using a multivariate binary logistic regression model that included variables such as age, sex, presence of cardiovascular risk factors, prior SARS-CoV-2 infection, and simultaneous administration of COVID-19 and influenza vaccines, we identified that delta values of anti-spike IgG antibody production at the three post-vaccination time points were independent predictors of the decremental response of endocan. These results confirmed the association between higher relative anti-spike IgG antibody production and lower endocan expression 24 days after vaccination. However, multivariate analysis revealed that the delta values of IgG antibody levels at all-time points were not sufficient to explain the heterogeneity between the incremental and decremental sVCAM-1 response.

## 4 Discussion

**Figure 3:**
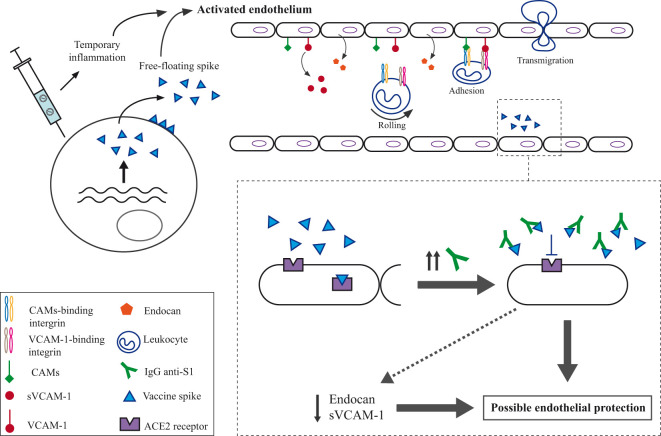
Illustration of the potential mechanism for the impact on the endothelium of the third dose of the BNT162b2 mRNA COVID-19 vaccine. Upper part: The vaccine induces both transient inflammation and spike protein generation, which would trigger endothelial activation. This endothelial activation might cause the release of endocan by endothelial cells and the proteolytic release of the soluble form of VCAM-1. Additionally, endocan release may promote the expression of cellular adhesion molecules (CAMs), such as VCAM-1, which play a critical role in mediating leukocyte adhesion to the vascular endothelium. By binding to integrins on the surface of activated leukocytes, VCAM-1 facilitates their firm adhesion to endothelial cells, enabling their migration across the endothelial barrier into surrounding tissues, where they contribute to the inflammatory response. Moreover, the free-floating vaccine spike could bind to the ACE2 receptor, resulting in some pro-thrombotic and inflammatory effects. Lower part: The IgG antibodies might potentially prevent this binding to ACE2, leading to a reduction in the secretion of endocan and sVCAM-1, which could ultimately protect the endothelium.

Two differential responses displayed by the endothelial markers, endocan and sVCAM-1, were identified from 7 to 90 days following administration of the third dose of the BNT162b2 mRNA COVID-19 vaccine. Consequently, participants were classified into two groups, each demonstrating either an increased or diminished response. The concurrent mild elevation in serum levels of endothelial markers among the incremental responders (34.29% of study subjects) over 90 days following mRNA booster vaccine administration may be attributed to temporary endothelial activation. Similar to our study, Ostrowski et al. (8) reported moderate increase in plasma concentrations of proteins related to endothelial function (VCAM-1, intercellular adhesion molecules and E-selectin) persisting for at least three months after the initial doses of both adenovirus and mRNA COVID-19 vaccines. A study demonstrating that mRNA COVID-19 vaccines cause a transient impairment of endothelial function, as assessed by flow-mediated dilation of the brachial artery (FMD) (9). This study showed a very short-term endothelial dysfunction in vaccinated individuals, which returned to baseline within 48 hours after the administration of the second dose. These findings could contrast with our data indicating participants who experienced a more prolonged elevation in endothelial markers, although endothelial function was measured using different techniques: FMD and ELISA assays. In line with this study’s findings, Yamaji et al. (20) very recently demonstrated that FMD, used to assess endothelial function, showed a temporary decline following the second dose of the BNT162b2 COVID-19 vaccine, with full recovery observed within six months. The mild activation of the endothelium observed in incremental marker responders might not be sufficient to cause endothelial damage, as the median post-vaccination levels of endocan and sVCAM-1 remained within the reference range for healthy controls reported in other studies (21–25). Nevertheless, it is important to note that in our participants (incremental responders) endocan and sVCAM-1 levels remained elevated for 90 days after vaccine administration. This prolonged endothelial activation, even if mild, could result in some endothelial injury. A prolonged activating stimulus may result in abnormal endothelial functioning and structural alterations, leading to inflammation mediated by the secretion of endothelial proteins, such as endocan and sVCAM, as well as a loss of vascular integrity. This loss of integrity is associated with endothelial cell detachment, which exposes a more thrombogenic extracellular matrix (26). Elevated endocan levels have been reported to promote inflammation in macrophages by inducing the expression of iNOS and C-reactive protein, as well as by increasing the production of nitric oxide and reactive oxygen species (27). Additionally, endocan stimulates endothelial cells and macrophages to secrete proinflammatory cytokines, including IL-1, TNF-α, and IL-6 (12, 28). As depicted in **Figure 3** (upper panel), endocan also induces the release of vascular adhesion molecules associated with inflammation, such as VCAM-1, from endothelial cells (28). This promotes the adhesion of circulating leukocytes to the vascular endothelium, thereby enhancing their recruitment and transendothelial migration into inflamed tissues (29). This should be taken into account in populations planned to receive multiple vaccine doses, especially in individuals with vascular risk factors. The persistent response induced by several vaccinations over time might have detrimental long-term effects on endothelial health. In fact, *in vitro* experiments have shown that continuous endothelial activation increases the expression of intracellular reactive oxygen species, a hallmark of endothelial dysfunction (30). The changes observed in the vascular endothelium following vaccination are not unique to COVID-19 vaccines. Over the past two decades, influenza vaccines have served as a model for mild immune system stimulation, based on a modest C-reactive protein response to injection (31–34). Despite causing only a slight elevation in C-reactive protein, which resolves completely within 14 days, influenza vaccination has been associated with abnormalities in arterial function, as measured by a decrease in flow-mediated dilation for at least 2 weeks after vaccination (35).

Interestingly, a significant proportion of participants (25.71%) experienced a simultaneous decrease in both biomarkers, reflecting a lower basal activation status of endothelial cells following mRNA vaccine administration. Statistical analysis revealed a significant positive association between higher delta values of IgG antibodies and reduced levels of endocan at 7, 15 and 24 days post-vaccination, as well as decreased levels of sVCAM-1 on day 7. These findings suggest that the reduced response of endothelial markers may be attributed to a greater relative increase in IgG antibody production, which could inhibit spike-ACE2 binding and thereby mitigate its harmful effects on the endothelium. It has been proposed that the binding of the spike protein to ACE2 results in a decrease in the enzymatic activity of this receptor, leading to an imbalance between angiotensin II and angiotensin 1-7, as well as the production of inflammatory mediators, oxidative stress, and endothelial dysfunction (36). We concur with the studies by Mogi (37), and Devaux and Camoin-Jau (10), which suggested that vaccine-generated antibodies may bind to free-floating spike protein, preventing its interaction with the ACE2 receptor. In fact, a recent study found that the detection of spike antigen in plasma after the second mRNA vaccine dose is impaired, probably due to the formation of circulating immune complexes of anti-spike antibodies and spike protein, reducing the detection and quantification of spike protein in plasma antigen recognition assays (38). In addition, several *in vitro* research (39) and on animal models (40) have provided evidence that the spike protein alone could have a direct negative impact on the endothelium (41). Thus, the inhibition of the interaction between spike and the ACE2 receptor would lead to endothelial protection and a decrease in the secretion of endothelial markers such as endocan and sVCAM-1 (as depicted in the lower part of **Figure 3**), resulting in a reduction of inflammation and leukocyte extravasation. In this context, the mechanisms by which higher delta values of IgG antibodies, indicating greater production of new anti-Spike IgG antibodies, might mitigate vaccine-induced endothelial damage could relate to differences in antibody affinity for the spike protein rather than total IgG levels, as we found no significant differences in total Ig levels post-vaccination among participants. Elevated pre-existing antibody levels may dampen the immune response to additional antigen exposure through a feedback inhibition mechanism, while individuals with low baseline IgG levels are likely to show a more substantial increase in new IgG antibody production (42). The newly produced antibodies may exhibit a higher affinity following a booster dose, as their immune systems become more responsive to antigen exposure and undergo robust affinity maturation. It should be noted that the hypothesis regarding the protective nature of anti-Spike IgG antibodies arises from the observation of two distinct groups exhibiting reduced and increased endothelial marker responses, which have not been reported previously. The previous studies have primarily demonstrated elevated levels of inflammatory, endothelial, and coagulation markers following vaccination, attributing endothelial activation to the indirect inflammatory effects of the vaccine (8, 9). Most of these studies analyze the whole cohort of participants and do not include intra-patient analyses to describe relative changes over an extended period post-vaccination. This absence of intra-patient analysis may explain the lack of findings suggesting potential endothelial protection conferred by vaccine-induced antibodies.

Regarding anti-spike IgG antibodies, the greatest relative increase in IgG antibody concentrations was observed in individuals without a history of COVID-19. In contrast, individuals with prior COVID-19 started with higher baseline IgG levels but experienced lower increases in IgG antibody production. These elevated pre-existing antibody levels may result from prior hybrid immunization through the first dose of vaccination followed by subsequent exposure to SARS-CoV-2, as previous studies by Stamatatos et al. (43) and Ontañón et al. (18) demonstrated that hybrid immunity significantly enhances the durability and breadth of neutralizing antibody responses. As discussed earlier, the findings of Dangi et al. (42) suggest that high pre-existing antibody levels may downregulate the immune response to the booster, which could explain the lower relative increases in antibodies observed. Another hypothesis for the decreased IgG delta in individuals with a history of COVID-19 could involve immune imprinting (also known as original antigenic sin). According to this phenomenon, the immune system’s initial or prior exposure to the virus’s spike protein shapes subsequent immune responses, potentially limiting the ability to respond effectively to modified spike-based vaccines. This could result in more moderate increases in IgG antibodies in such participants, leading to increased endothelial activation. Therefore, healthy individuals without pre-existing medical conditions, previously infected with SARS-CoV-2, and exhibiting elevated baseline levels of spike-specific IgG may not require a COVID-19 booster dose as urgently, or could potentially receive it at extended intervals after prior doses. In addition, our analysis, in agreement with previous studies (40, 41), found no significant differences in serum levels of spike-specific IgG antibodies between participants who received only the COVID-19 vaccine and those with simultaneous COVID-19 and influenza inoculations. Hence, our results would suggest that measuring specific biomarkers before vaccination, such as spike-specific IgG antibody levels and endothelial markers, particularly endocan, which correlates with antibody levels within 24 days post-vaccination, could potentially predict individual responses to the vaccine. The observed association between higher relative increases in antibodies and a reduction in endothelial activation markers among decremental responders highlights the potential for using these biomarkers to identify individuals who might experience either a more protective or an adverse endothelial response following vaccination. If similar patterns are confirmed in more diverse populations, including those at higher risk for adverse vaccine events or with varying demographic profiles, tracking these biomarker levels could serve as a valuable tool for predicting and managing vaccine-related risks, especially in individuals with underlying health conditions. This approach could assist in tailoring booster dose recommendations and developing preventive strategies for those at higher risk of vascular complications.

However, some limitations should be taken into account. In our study, we focused on a small, predominantly female cohort of healthy, middle-aged individuals, and did not include participants at increased risk of adverse events following vaccination. Thus, the generalizability of these findings to more diverse populations, including older adults or individuals with underlying cardiovascular, autoimmune, thrombotic or inflammatory conditions, remains uncertain. In addition, although we present data on IgG against RBD of the spike protein, indicating that the third dose increases antibody levels, the measurement of neutralizing antibodies, along with the analysis of inflammation and coagulation biomarkers, was not possible due to insufficient volume of serum samples and the unavailability of PBMCs. These measurements could have offered more precise insights into the protective effects of vaccine-induced immunity. However, it is worth noting that several studies have demonstrated a strong correlation between anti-RBD spike antibodies and neutralizing antibodies (44, 45), as well as the relationship between humoral and cellular immunity following vaccination (46).

In conclusion, administration of the COVID-19 booster vaccine with BNT162b2 mRNA resulted in different response patterns for markers of endothelial function among study participants. A decrease in endothelial biomarker level was significantly associated with a higher relative production of anti-spike IgG antibodies during the 24-day post-vaccination follow-up period. This antibody response may be influenced by previous COVID-19. The present study contributes to the understanding of the endothelial response to the BNT162b2 mRNA vaccine, which could be critical for informing decisions regarding the need and timing of booster doses and for implementing prophylactic therapies to avoid undesirable thrombotic and/or inflammatory events in vaccinated individuals. Further studies providing a long-term assessment of both endothelial activation, measured by endocan levels, and neutralizing IgG antibody titers in larger and more diverse populations, including high-risk groups, could strengthen the robustness of the results and clarify the broader applicability of our findings.

## Data Availability

The original contributions presented in the study are included in the article/**Supplementary Material**. Further inquiries can be directed to the corresponding authors.
