# Supplementary material for: Distinct response patterns of endothelial markers to the BNT162b2 mRNA COVID-19 booster vaccine are associated with the spike-specific IgG antibody production
Source: Front Immunol. 2025 Jan 6;15:1471401. doi: 10.3389/fimmu.2024.1471401 (PMC11743620; doi:10.3389/fimmu.2024.1471401)
Supplement: Supplementary file 1 [file Table1.docx]

Supplementary Material

# Supplementary Tables

**Supplementary Table 1 (Table S1).** Demographic and clinical data of the study population.

| **Number subjects** | 38 |
| --- | --- |
| **Age (years)** | 48.11 ± 10.77 |
| **Female, n (%)** | 30 (79) |
| **Cardiovascular risk factors** |  |
| Diabetes, n (%) | 2 (5.26) |
| Dyslipidemia, n (%) | 6 (15.79) |
| Hypertension, n (%) | 5 (13.16) |
| Auricular fibrillation, n (%) | 0 (0) |
| **Previous COVID-19, n (%)** | 17 (44.74) |
| **Co-administration of COVID-19 and influenza vaccination, n (%)** | 25 (65.7) |
| **Pharmacological treatment** |  |
| Antihypertensive, n (%) | 3 (7.89) |
| Antidiabetic agent, n (%) | 1 (2.63) |
| Statins, n (%) | 2 (5.26) |
| Anti-inflammatory, n (%) | 2 (5.26) |

**n:** analyzed subjects.

**Supplementary Table 2 (Table S2).** Comparison of delta values of endothelial marker levels between previously infected and non-infected subjects within either incremental or decremental response groups.

|  |  | **Past COVID-19** | | |
| --- | --- | --- | --- | --- |
|  | **Delta** | **Infected** | **Non-infected** | **p** |
| **Incremental-responders (median [IQR],n)** | **Endocan 7d** | 0.172 [0.1962],8 | 0.2995 [0.7686],11 | 0.8404 |
|  | **Endocan 15d** | 0.3718 [0.2656],7 | 0.7458 [0.4975],8 | 0.0933 |
|  | **Endocan 24d** | 0.1439 [0.214],8 | 0.4069 [0.2493],8 | 0.1304 |
|  | **Endocan 90d** | 0.2341 [0.1472],5 | 0.5366 [0.7934],13 | 0.7028 |
|  | **sVCAM-1 7d** | 0.2277 [0.5949],10 | 0.3138 [0.4204],13 | 0.7844 |
|  | **sVCAM-1 15d** | 0.1849 [0.391],11 | 0.1992 [0.4353],10 | 0.5573 |
|  | **sVCAM-1 24d** | 0.431 [0.3331],9 | 0.3343 [0.3335],10 | 0.549 |
|  | **sVCAM-1 90d** | 0.168 [0.1785],6 | 0.3139 [1.062],9 | 0.607 |
| **Decremental-responders (median [IQR],n)** | **Endocan 7d** | -0.3362 [0.3187],8 | -0.3046 [0.1387],9 | 0.9858 |
|  | **Endocan 15d** | -0.2746 [0.1008],6 | -0.2558 [0.2562],11 | 0.7016 |
|  | **Endocan 24d** | -0.2626 [0.2589],6 | -0.2791 [0.1017],11 | 0.9655 |
|  | **Endocan 90d** | -0.2704 [0.279],5 | -0.2426 [0.072],4 | 0.4029 |
|  | **sVCAM-1 7d** | -0.3662 [0.3899],7 | -0.2966 [0.1652],7 | 0.7853 |
|  | **sVCAM-1 15d** | -0.2332 [0.1495],3 | -0.1477 [0.373],9 | 0.7273 |
|  | **sVCAM-1 24d** | -0.3335 [0.2541],6 | -0.2784 [0.3121],9 | 0.7296 |
|  | **sVCAM-1 90d** | -0.5046 [0.2995],7 | -0.1645 [0.4427],8 | 0.3418 |

**Supplementary Table 3 (Table S3).** Delta values of spike-specific IgG antibodies concentrations among endocan and sVCAM-1 response groups.

|  | **Endocan** | | | **sVCAM-1** | | |
| --- | --- | --- | --- | --- | --- | --- |
|  | **Incremental response group** | **Decremental response group** | **p** | **Incremental response group** | **Decremental response group** | **p** |
| **Delta IgG 7d (median [IQR],n)** | 7.885 [14.64],16 | 40.73 [54.07],18 | 0.0224 | 7.085 [31.67],22 | 30.03 [45.78],13 | 0.0487 |
| **Delta IgG 15d (median [IQR],n)** | 7.077 [30.45],16 | 43.93 [55.59],19 | 0.024 | 9.561 [39.78],23 | 38.58 [51.48],13 | 0.1311 |
| **Delta IgG 24d (median [IQR],n)** | 6.396 [21.01],13 | 53.91 [42.84],11 | 0.0257 | 6.396 [39.93],15 | 36.73 [46.5],10 | 0.1151 |
| **Delta IgG 90d (median [IQR],n)** | 2.893 [12.57],16 | 12.81 [83.34],19 | 0.0883 | 4.62 [33.8],23 | 12.81 [30.45],13 | 0.2528 |
